# A Report of a Rare Case of an Advanced Adult Granulosa Cell Tumor Initially Diagnosed as Primary Ovarian Melanoma

**DOI:** 10.7759/cureus.7921

**Published:** 2020-05-01

**Authors:** Rania Chacha, Achraf Miry, Badr Serji, Sami Aziz Brahmi, Said Afqir

**Affiliations:** 1 Oncology, Mohammed VI University Hospital, Oujda, MAR; 2 Pathology, Mohammed VI University Hospital, Oujda, MAR; 3 Surgical Oncology, Mohammed VI University Hospital, Oujda, MAR; 4 Medical Oncology, Mohammed VI University Hospital, Oujda, MAR

**Keywords:** primary ovarian melanoma, granulosa cell tumor, case report, morocco

## Abstract

Ovarian granulosa cell tumors are rare gynecological cancers with favorable clinical evolution and survival outcomes. We report a new case of this presentation in a patient that was initially diagnosed as a bilateral primary melanoma of the ovary. The patient is a 51-year-old woman with a history of abdominal swelling and deterioration of her general conditions. Physical examination revealed abdominal distension and diffuse dullness with initially highly elevated cancer antigen 125. Contrast-enhanced thoracoabdominal-pelvic computed tomography showed a left-sided ovarian mass and abundant ascites and pleurisy. Ex-lap surgery found two large bilateral ovarian masses associated with peritoneal carcinomatosis and highly abundant ascites. The histopathological examination of the omental biopsy revealed an undifferentiated tumor proliferation of cells with highly positive Human Melanoma Black 45 marker in favor of an achromic malignant melanoma according to the pathologist. Because of her advanced disease, the patient received a combination of six cycles of neoadjuvant dacarbazine, cisplatin, and paclitaxel and showed partial response based on the response evaluation criteria in solid tumors, followed by total abdominal hysterectomy and bilateral salpingo-oophorectomy with cytoreductive surgery. Unexpectedly, the histopathological analysis of the surgical specimens was in favor of an advanced adult granulosa cell tumor with positive inhibin B. Our patient is alive at her 13^th^ month of survival and is being followed by the oncology team. The challenges of the pathological diagnosis of this case are discussed. The diagnosis of primary ovarian melanoma should not be based on one immunohistochemical marker only. A single biopsy of omental implants in peritoneal carcinomatosis during ex-lap surgery should be avoided.

## Introduction

Malignant melanoma of the gynecologic tract is rare with a particular aggressive behavior and poor survival outcomes [1]. Primary ovarian melanoma (POM) is rarer and few published cases reported this unusual anatomical location. In most cases, POM arises as a result of the malignant transformation of the melanocytes of the ectoderm of an ovarian cystic teratoma [2]. Surgical biopsy is the standard procedure for diagnosis and staging. Usually, the histopathological review of POM is based on at least two markers including Human Melanoma Black 45 (HMB-45) and Melan A. Adjuvant and neoadjuvant chemotherapy (NACT) combining paclitaxel, cisplatin, dacarbazine, and other anticancer drugs in addition to surgery may be delivered to improve outcomes in POM patients. Sometimes, the diagnosis of this entity may be erroneous if the surgical biopsy was not well performed particularly without covering the primary tumor, in addition to, the incompleteness of the pathological investigation based on a single melanoma biomarker such as HMB-45. This marker can be positive in other disease conditions such as tumors infiltrated by siderophages. This challenging situation is reported in our paper which describes a case of an advanced adult granulosa cell tumor that was initially diagnosed as POM. The clinical, pathological, and therapeutic findings of this challenging case are reported as recommended by the recently launched CARE guidelines [3].

## Case presentation

**Figure 1:**
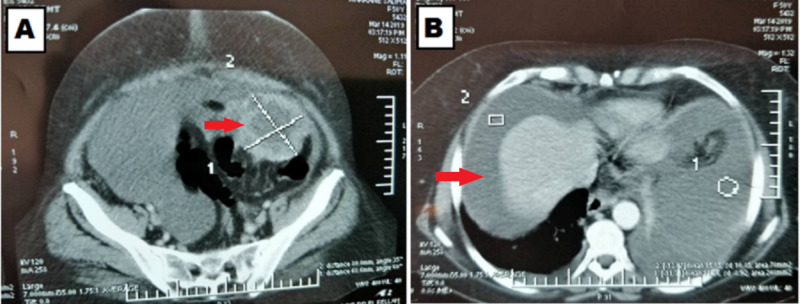
An image illustrating the initial diagnostic contrast-enhanced thoracoabdominal-pelvic computed tomography showing a left-sided ovarian mass (A) and abundant pleural effusion (B).

Our patient was a 51-year-old white woman from the Rif region of Morocco (Amazigh ethnicity) that presented abdominal swelling and deterioration of her general conditions three months before her visit to a general practitioner. The patient was previously diagnosed with hypertension and is currently under angiotensin-converting enzyme inhibition without any family history of hereditary cancer or other diseases. Her physical examination revealed abdominal distension and diffuse dullness with initially highly elevated cancer antigen 125 (CA-125) at 461.7 U/mL. Diagnostic imaging based on contrast-enhanced thoracoabdominal-pelvic computed tomography (TAP-CT) (Figure 1) showed a left-sided ovarian mass and abundant ascites and pleurisy. 

**Figure 2:**
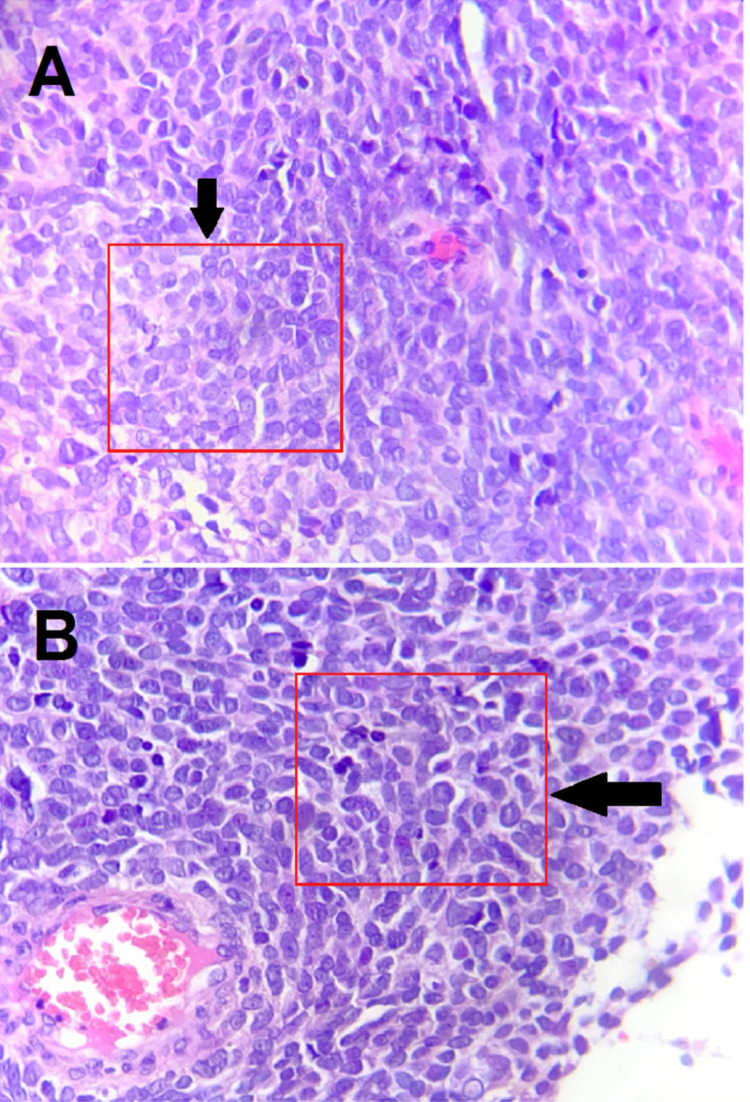
The omental tissue was largely replaced by sheets of malignant tumors on surgical biopsy (A). Cytonuclear atypia were mild (HE; 400X) (B). HE: Hematoxylin and eosin stain.

The patient was then referred to our department for specialized management. Given the strong suspicion of advanced ovarian cancer, ex-lap diagnostic surgery was performed. During surgery, two large bilateral ovarian masses associated with peritoneal carcinomatosis and highly abundant ascites (approximately 4 L) but without liver and spleen dissemination were found upon exploration and an omental biopsy was performed. The postoperative course was uneventful. Microscopically (Figure 2), the sections examined found an omental tissue infiltrated by an undifferentiated tumor proliferation made of layers of cells with unclear cytoplasmic limits. These tumor cells have a rounded or oval nucleus with slightly mottled chromatin. Cytonuclear atypia was mild to moderate and there were few mitoses.

**Figure 3:**
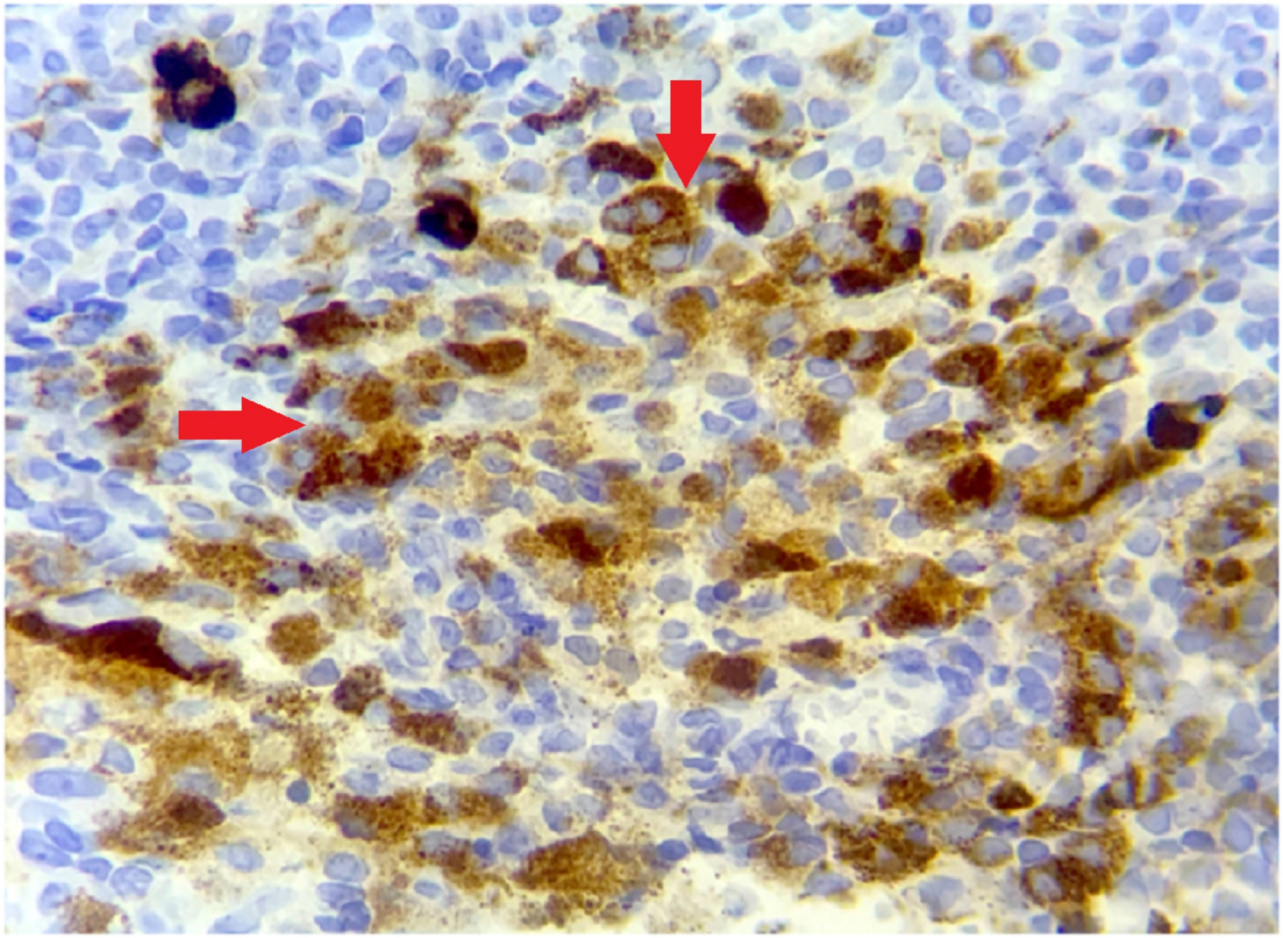
Tumor cells express HMB-45 marker on surgical biopsy (HE; 400X). HMB-45: Human melanoma black; HE: Hematoxylin and eosin.

Immunohistochemistry (IHC) based on 13 antibodies covering the most known possible tissue origins of tumor infiltration was used for differential diagnosis on surgical biopsy (Table 1). The IHC analysis was in favor of a secondary infiltration of an achromic malignant melanoma with positive staining of HMB-45 marker (Figure 3). After the surgical biopsy was performed which confirmed the melanocytic nature of the tumor according to the local pathologist, no foci of primary malignant melanoma except for the ovary were found upon dermatological examination.

**Table 1:** A summary of the initial immunostaining findings of the omental biopsy. CK: Cytokeratin; IN-α: Inhibin alpha; P53: Tumor protein 53; VIM: Vimentin; ASM: Anti-smooth muscle; S-100; Protein S-100; Ki67: Kiel-67 protein; HMB-45: Human melanoma black 45; WT1: Human Wilms' tumor.

Antibodies used	Staining
Anti-Calretinin	Negative
Anti-CK AE1/AE3	Negative
Anti-CK20	Negative
Anti-CK7	Heterogeneous and moderate positivity of some clusters of tumor cells
Anti-INα	Negative
Anti-P53	Negative
Anti-VIM	Negative
Anti-WT1	Negative
Anti-ASM	Negative
Anti-Desmin	Negative
Anti-S100	Negative
Anti-HMB-45	Heterogeneous and high positivity of some clusters of tumor cells
Anti-Ki67	Positive (estimated at 10%)

**Figure 4:**
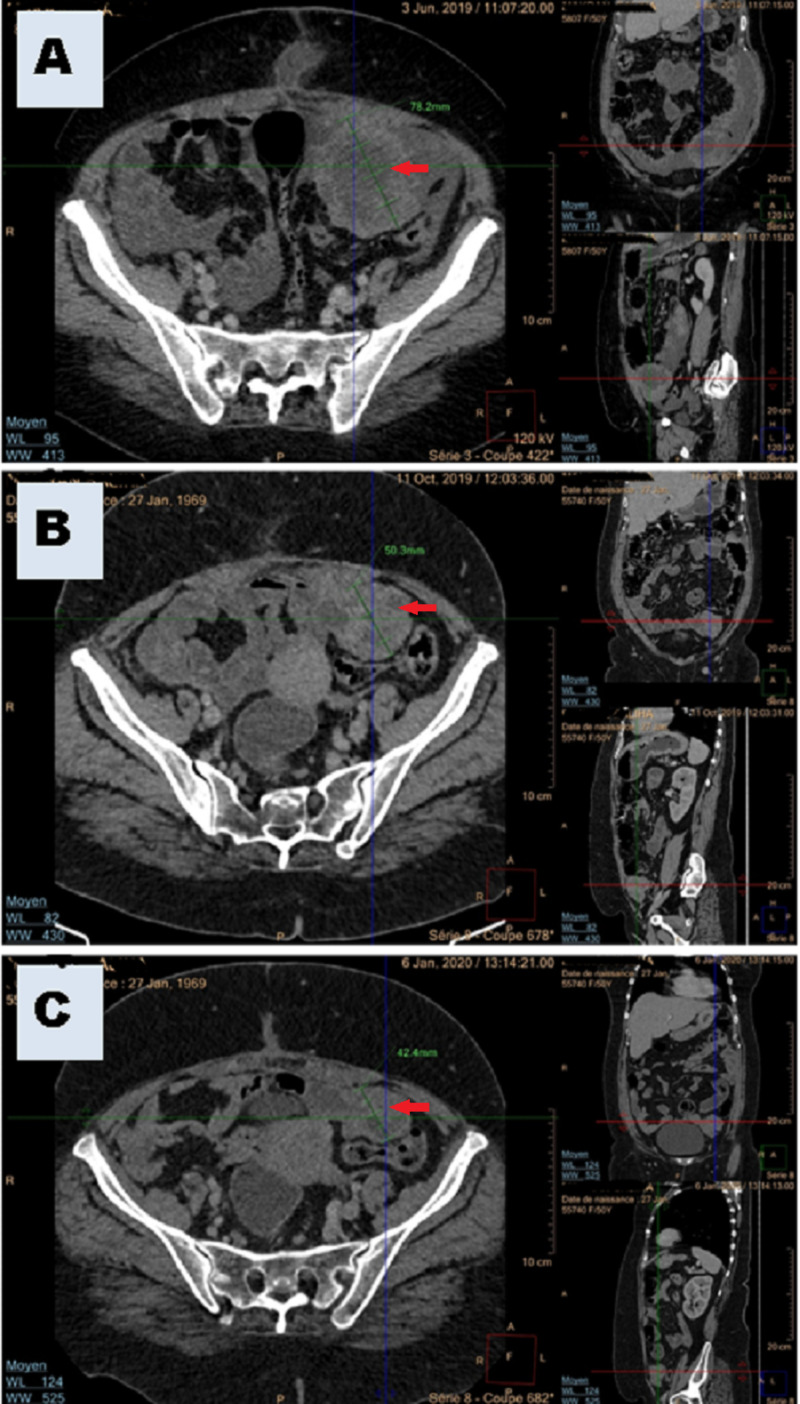
Follow-up imaging findings after (A) diagnostic ex-lap, (B) after six months of follow-up (after ex-lap and third cycle of neoadjuvant chemotherapy), (C) one month before hysterectomy and cytoreductive surgery.

Given the advanced disease, the patient received a combination of neoadjuvant dacarbazine (DTIC, 800 mg/m^2^; day 1), cisplatin (CDDP, 20 mg/m^2^; days 1, 2 and 3), and paclitaxel (PTX, 80 mg/m^2^; days 1 and 8) every three weeks. After six months of NACT, there was a decrease in the size of the known left ovarian tumor (52 × 49 mm against 91 × 77 mm) in favor of a partial tumor response (Figure 4A-4B). Moreover, a complete regression of pleural effusion and peritoneal carcinomatosis and a marked decrease of CA-125 (9.19 U/mL against previous initial 461.7 U/mL) were also noted. The patient continued her treatment under NACT until the sixth cycle and she was reevaluated. TAP-CT showed a partial response (Figure 4C) (51 × 37 against 52 × 49 mm) without the appearance of new metastases. During the third cycle of NACT, accidental drug extravasation occurred in the left forearm and it was successfully managed with topical corticosteroids. The patient tolerated NACT well without any unanticipated adverse events that require reducing dose schedule or changes in interventions.

**Figure 5:**
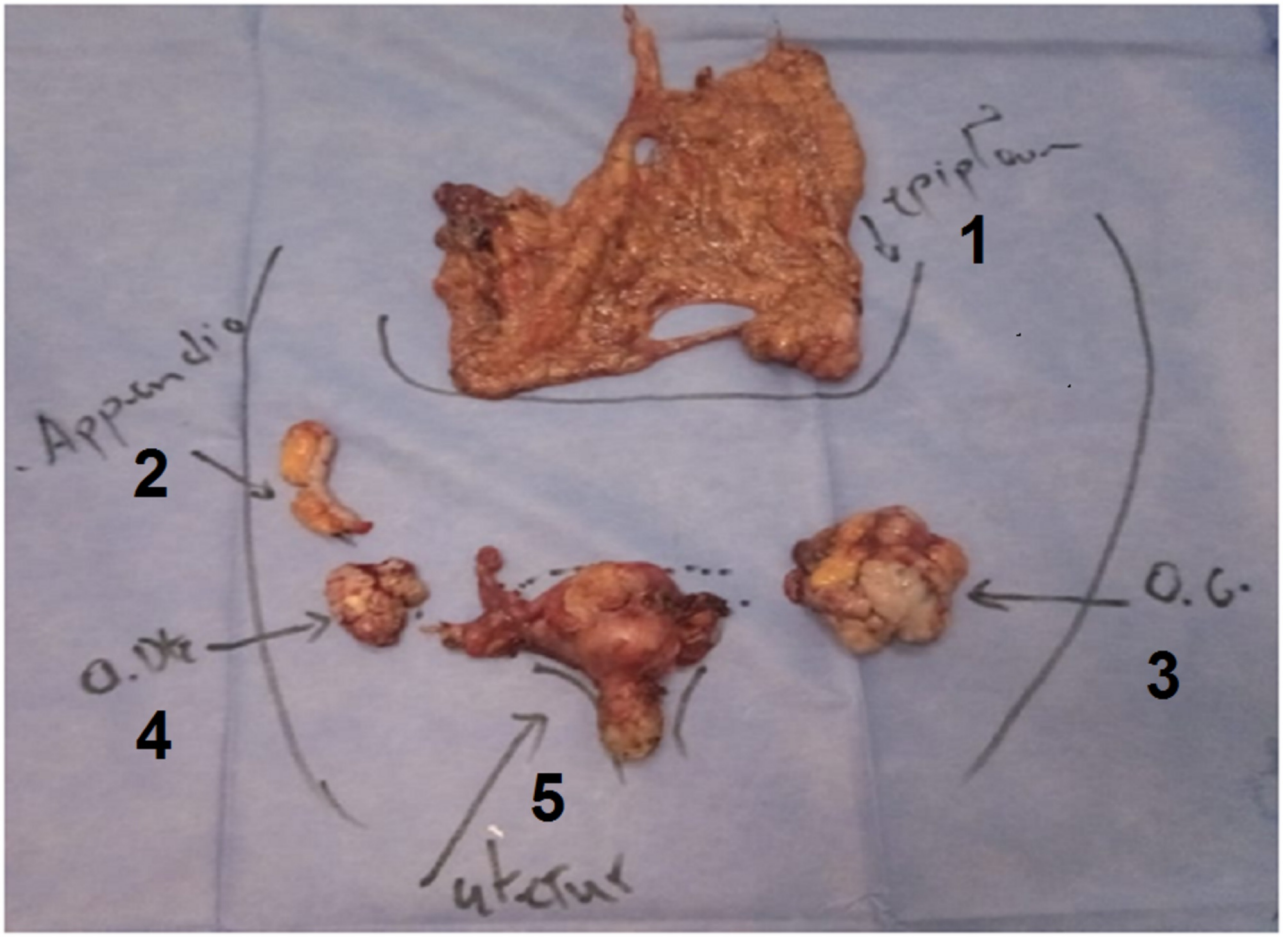
Surgical specimens of total hysterectomy, salpingo-oophorectomy, appendectomy, and omentectomy. 1: omentum; 2: appendix; 3: left ovary; 4: right ovary; 5: uterus.

Following the multidisciplinary tumor board meeting, total abdominal hysterectomy and bilateral salpingo-oophorectomy with cytoreductive surgery were indicated (Figure 5).

Surprisingly, the final pathological examination of all surgical specimens by an expert pathologist based on a panel of several markers was in favor of an adult granulosa cell tumor with negative HMB-45 and positive Inhibin B marker (Table 2). The other tumor markers of melanoma were also negative. Adjuvant chemotherapy based on bleomycin, etoposide, and cisplatin (BEP) combination is being programmed. Our patient remains alive at the time of this case writing with 13 months of overall survival.

**Table 2:** A summary of the final immunostaining report of the tissue specimens of bilateral salpingo-oophorectomy. CK: Cytokeratin; HMB-45: Human melanoma black 45; PS100: Protein S100.

Antibodies used	Staining
Anti-CK	Negative
Anti-Melan A	Negative
Anti-HMB-45	Negative
Anti-PS100	Negative
Anti-inhibin B	Positive

## Discussion

Primary and secondary melanomas of the ovary are rare tumors that were described only in historical case reports. Malignant transformation of teratomas is believed to induce histological differentiation into squamous cell carcinoma (88%) and other minor types including POM [4]. Given the absence of international guidelines, the management of these cancer patients is difficult and is based only on the published experiences. We reviewed the previous literature before the re-diagnosis and we used a dacarbazine-based chemotherapy which provided a partial response in our case [5].

Surgery is the cornerstone for treating ovarian melanomas [4]. Total hysterectomy and bilateral salpingo-oophorectomy and debulking are proposed to post-menopausal patients and conservative surgery is an option to preserve fertility for young premenopausal women. Surgery alone showed the best overall survival of four years [6]. NACT may be proposed for patients with morbid conditions and peritoneal carcinomatosis as in our patient. The chemotherapeutic regimen containing dacarbazine used for treating our patient seems to be active also on adult granulosa cell tumor. To our knowledge, this was not previously reported and the main treatment of these gynecological malignancies is still based on the BEP protocol [7]. An important observation in our case is the fact that the tumor was unusually bilateral. In most cases (95%), adult granulosa cell tumors arose in a unilateral manner [8,9]. The advanced and aggressive behaviors of the tumor of our patient may be due to the bilaterality of its location. It is well known that this phenotype is associated with advanced disease at presentation [9]. Our patient was initially diagnosed with bilateral POM with a similar clinical presentation of that of primary epithelial ovarian cancer with positive HMB-45 staining on the initial biopsy examination. Overall survival after dacarbazine-based NACT and surgery was remarkably improved. After debulking surgery, the patient was re-diagnosed with another entity. The final expert report showed that this marker is not expressed in all resected specimens. When reviewing the literature, it appears that HMB-45 staining should be performed in association with other melanoma markers such as Melan A. This highlights the urgent need of expert pathologists in this field as well as the development of molecular testing in low-income settings. HMB-45 may be false-positive such as in the case of tumor-infiltrating siderophages that may express this melanoma marker as well. We decided to publish this case for the important educational messages for young oncologists and pathologists. The patient clinical evolution was well and her treatment is being switched to the recommended chemotherapy protocol.

## Conclusions

In this paper, we reported a new case of adult granulosa cell tumor of the ovary that was first diagnosed as bilateral POM. Importantly, we recommend to all surgical oncologists to avoid surgical biopsies for diagnostic purposes on peritoneal implants. The primary tumors should be targeted for biopsy. Moreover, the pathologists should use more than one marker to suggest melanoma in their pathological review. Of note, the association of surgery and chemotherapy combining cisplatin, paclitaxel, and dacarbazine demonstrated clinical response in adult granulosa cell tumor of the ovary.
